# The traits that predict the magnitude and spatial scale of forest bird responses to urbanization intensity

**DOI:** 10.1371/journal.pone.0220120

**Published:** 2019-07-25

**Authors:** Grant D. Paton, Alexandra V. Shoffner, Andrew M. Wilson, Sara A. Gagné

**Affiliations:** 1 Department of Geography and Earth Sciences, The University of North Carolina at Charlotte, Charlotte, North Carolina, United States of America; 2 Department of Fisheries and Wildlife, Michigan State University, East Lansing, Michigan, United States of America; 3 Environmental Studies Department, Gettysburg College, Gettysburg, Pennsylvania, United States of America; INIBIOMA (Universidad Nacional del Comahue-CONICET), ARGENTINA

## Abstract

As humans continue moving to urban areas, there is a growing need to understand the effects of urban intensification on native wildlife populations. Forest species in remnant habitat are particularly vulnerable to urban intensification, but the mechanisms behind these effects are poorly understood. An understanding of how species traits, as proxies for mechanisms, mediate the effects of urban intensification on forest species can help fill this knowledge gap. Using a large point count dataset from the Second Pennsylvania Breeding Bird Atlas, we tested for the effects of species traits on the magnitude and spatial scale of the responses of 58 forest bird species to urbanization intensity in landscapes surrounding count locations. Average urbanization intensity effect size across species was -0.36 ± 0.49 (SE) and average scale of effect of urbanization intensity was 4.87 ± 5.95 km. Resident forest bird species that are granivorous or frugivorous, cavity-nesting, and have larger clutch sizes and more fledglings per clutch had more positive associations with increasing urbanization intensity in landscapes. In addition, the effect of urbanization intensity on forest birds manifested most strongly at larger spatial scales for granivorous, frugivorous, or omnivorous species that are cavity-nesting, have larger clutch sizes and longer wingspans, and flock in larger numbers. To our knowledge, the present study represents the first direct tests of the effects of species traits on both the magnitude and spatial scale of the effect of urbanization on forest birds, as well as the first evidence that migratory status, clutch size, wingspan, and fledglings per clutch are important determinants of the responses of forest birds to urbanization. We discuss the possible mechanisms underlying our results and their implications for forest bird conservation in urbanizing landscapes.

## Introduction

The 21^st^ century will be defined by the expansion of urban development. Fifty-four percent of the world’s population lived in urban areas in 2014, with 66% expected to be doing so by 2050 [1]. As a result, total urban area is projected to grow by at least 430,000 km^2^ from 2000 to 2030, an increase of 139% [2]. Expanding urban centers have significant impacts on species in remnant habitat, such as forest breeding birds. The abundance of generalist species in forest patches is affected by proximity to and intensity of surrounding urban development [3, 4, 5]. For example, blue jay (*Cyanocitta cristata*) abundance is over three times greater in forests surrounded by medium-intensity housing than in those surrounded by low-intensity housing [6]. Urbanization also has particularly strong negative effects on the abundances of specialist and forest interior species [7, 8, 9, 10]. For example, based on data from the French Breeding Bird Survey, populations of specialist species become more unstable and prone to collapse as urbanization increases [11].

The mechanisms by which urbanization affects forest bird species include reduced movement success, altered resource availability, and increased disturbance [12]. More intense urbanization increases the effective isolation of forest patches [13, 14] and likely increases dispersal mortality by means of collisions with buildings and vehicles, and predation by domestic cats [15, 16]. Conversely, urban areas can be a source of additional resources to birds in forest patches, favoring species that can take advantage of these opportunities [17, 18, 19]. However, the replacement of native habitat with developed land may make native food sources rarer, imperiling species that have specific dietary or nesting needs [20]. Finally, urban development is associated with increases in anthropogenic noise, air temperature, pollution, parasitism, and predation, all of which significantly impact habitat quality for birds [21, 22, 23, 24, 25, 26, 27, 28].

Species traits are useful in elucidating the importance of these mechanisms in structuring forest bird communities. Forest bird traits found to be associated with high urban abundances highlight the altered resource availability and/or increased disturbance of urban settings: an omnivorous diet, a medium or high nesting height, sociality, biparental involvement in nest construction, long duration in nest after hatching, and cavity nesting [8, 20, 29]. Omnivory may indicate a more generalized feeding behavior, potentially enabling a forest species to utilize anthropogenic food sources, such as trash, non-native vegetation, and bird feeders and/or feeding tables [30]. Medium to high nesting heights may ensure that forest bird nests are less exposed to disturbances from human activity [31, 32] and nest predation [33, 34]. Cavity nesting may also similarly reduce the exposure of nests to human disturbance and predation [33]. Species that live in medium- or large-sized groups may benefit from urban survival strategies shared among group members [35] and biparental involvement in nest construction may result in more stable nests that can better withstand urban disturbances [36]. Species that spend longer periods of time in the nest before fledging require lower daily resource needs than faster-growing species, making them more suited to a potentially resource-depleted urban area [20, 37].

Despite these insights, there is much to gain in our understanding of forest bird trait variation associated with urbanization. First, we are not sure whether traits associated with urbanization success in birds in general, such as being a resident rather than a migratory species, large clutch size, multiple clutches per year, and large body size, wingspan, and brain size [20, 38, 39, 40, 41, 42, 43], also characterize abundant forest bird species in urban landscapes. Particularly lacking is evidence of associations between forest bird success in urban landscapes and traits that infer differential movement success, such as wingspan. While prior research on birds has included some forest species in analyses, this is the first study to our knowledge to focus specifically on the assemblage. Second, we have no direct evidence that traits characterizing abundant forest bird species in urban landscapes are in fact associated with positive responses to urbanization. In other words, no study to date has statistically tested the effects of species traits on the responses of forest birds to urbanization. Third, we do not know how species traits influence the spatial scale at which urbanization affects forest birds despite the likelihood that mechanisms underlying the effects of urbanization operate at a variety of spatial scales [44].

The objective of the present research was to begin to address these three gaps in our knowledge of forest bird trait variation in urban landscapes. Using a large point count dataset from the Second Pennsylvania Breeding Bird Atlas, we first estimated the effect of urbanization intensity in landscapes of multiple scales on the individual occurrences of 58 forest bird species, controlling for other potentially important explanatory variables such as forest amount and configuration, local habitat quality, and species detectability. For each species, we selected the coefficient for urbanization intensity with the largest absolute magnitude, and the spatial scale at which this occurred. These data were then used as the response variables in a second set of analyses that used species traits as predictor variables. Traits represented species characteristics linked to the mechanisms by which urbanization affects forest birds, such as: a greater capacity for movement leading to high movement mortality and reduced movement success (wingspan, migratory status, and territory size); differential resource affinity or need for resources for growth and reproduction (body size, dietary habit, foraging height, clutch size, number of fledglings per clutch, number of clutches per year, and duration in the nest); and differential vulnerability to disturbance agents such as domestic cats and anthropogenic noise (nesting height, parental involvement in nest construction, cavity nesting, flock size, lifespan, and song attributes). To our knowledge, this is the first study to use this approach.

## Materials and methods

### Study area

**Fig 1:**
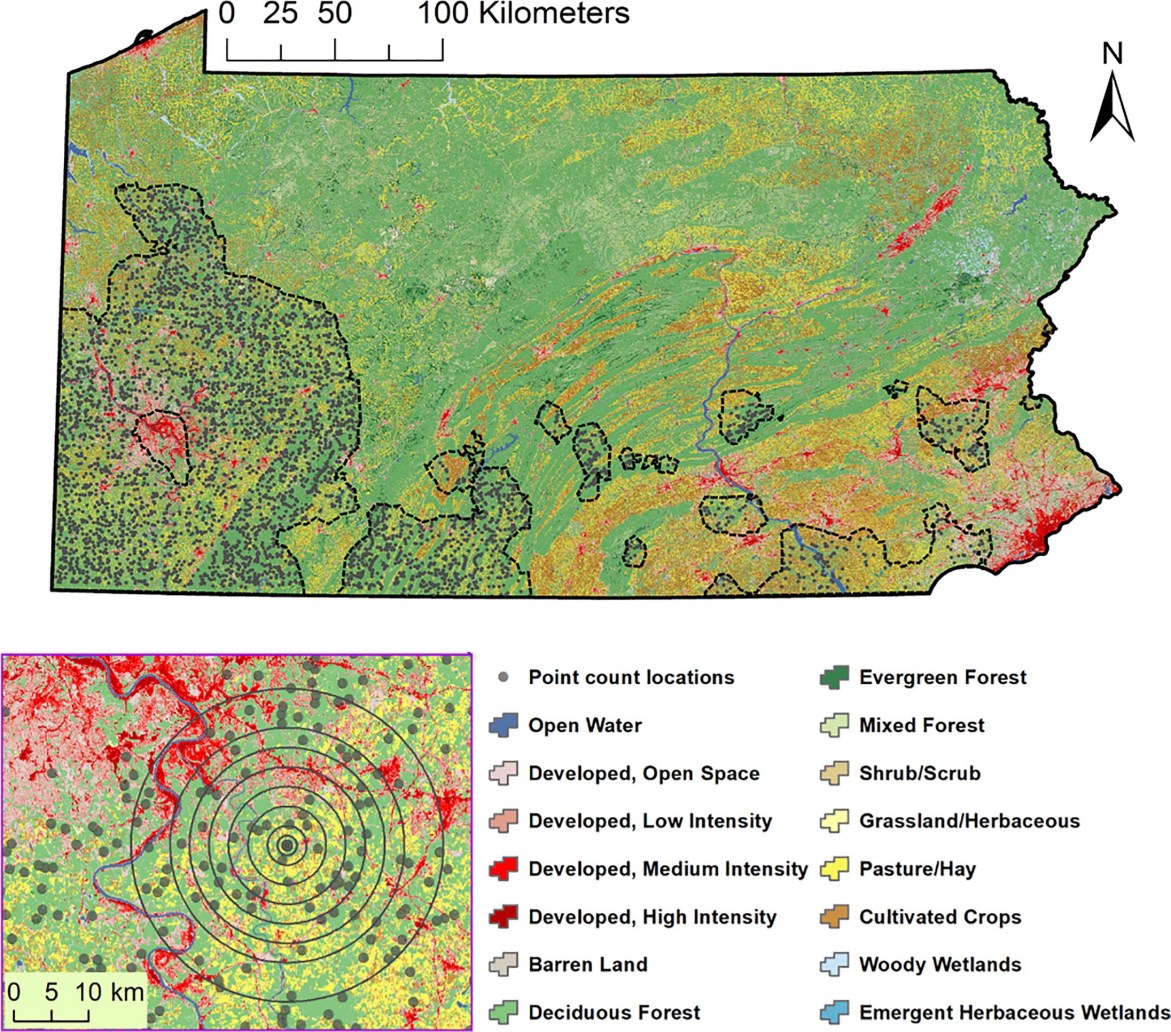
Second Pennsylvania Breeding Bird Atlas point count locations within the estimated range, delineated by dashed lines, of an example species, the Kentucky Warbler (*Geothlypis formosa*). The inset depicts landscapes of multiple spatial scales centered on a count location. Land cover is from the 2006 National Land Cover Database [51].

Pennsylvania is approximately 120,000 km^2^ in area between 39.0° and 42.5°N and 74.0° and 81°W in the northeastern United States (Fig 1). Elevations are typically 300 to 400 m, except where the state is bisected by the Appalachian and Allegheny mountain ranges (maximum elevation = 980 m), and the coastal southeastern corner of the state [45]. The climate is Humid Continental in the northern half of the state and along the Appalachian Mountains and Humid Subtropical elsewhere [46]. Pennsylvania is predominantly covered by forest– 58% of total land area [47]–dominated by red (*Quercus rubra*) and white oak (*Quercus alba*), with agricultural activity occurring in the lower elevations [48]. Average population density is 100 people/km^2^, with roughly 65% of the population residing in the Philadelphia or Pittsburgh metropolitan areas [49]. By 2040, Pennsylvania’s population is expected to grow by 11% to approximately 14.1 million people, with most of the growth expected in the Philadelphia metropolitan area and the center of the state [50].

### Second Pennsylvania Breeding Bird Atlas

The Second Pennsylvania Breeding Bird Atlas (PBBA) was a formal breeding bird survey conducted statewide between 2004 and 2009 in a grid of 4,937 blocks, each approximately 25 km^2^ in area [52]. Within each survey block, trained staff conducted up to eight point counts, depending on block size and road accessibility for a total of 33,763 counts in the state. Some completed surveys were omitted from the final analysis due to quality control measures. Count locations were selected by random placement of a point within a block, with adjustments made to locate points on the nearest non-highway road. Point locations across the state were at least 400 m apart. Each point was surveyed once within the 5-year PBBA period in the spring or summer between May 25 and July 4. Twenty-two paid observers received extensive training before each field season. Observers recorded the species of each bird heard or seen within an unlimited distance from the point for 6 minutes and 15 seconds on weekday or weekend mornings, between 5 and 10 am, provided there were no adverse weather conditions.

### Forest bird species

From the 176 species detected during PBBA point counts, we excluded species that had the following characteristics: 1) hybrid species; 2) irregular breeder in the state; 3) raptor; 4) waterfowl; 5) nocturnal species and 6) occurrence at fewer than 30 count locations. The forest dependency of each of the remaining 101 species was assessed by comparing the cumulative distribution of counts of the species, across all 33,763 count locations, to the cumulative distribution of forest amount in landscapes at each scale using Kolmogorov-Smirnov tests of no difference (S1 Fig). If a large proportion of the individuals of a species occurred in the landscapes with the most forest or if individuals of a species accumulated in landscapes at the same rate that forest cover increased in landscapes, then the species was classified as "forest-dependent" (S1 Table). Otherwise, the species was classified as “forest-independent”, i.e., if a large proportion of the individuals of a species occurred in the landscapes with the least forest (S1 Table).Two species, the Black-billed Cuckoo (*Coccyzus erythrophthalmus*) and the Yellow-rumped Warbler (*Setophaga coronata*), were too rare to permit estimation of the effect of urbanization intensity on their occurrences, resulting in a final count of 58 forest-dependent species.

### Landscape definition and selection

We defined landscapes as circular areas centered on point count locations that were surrounded by >50% forest cover–composed of the Deciduous, Mixed, and Evergreen Forest classes of the 2005 National Land Cover Database (NLCD) [51]–within a 0.2 km radius (Fig 1). Deciduous forest was defined as forest cover in which deciduous species accounted for 75% or more of the cover. Evergreen forest was defined as forest cover in which evergreen species accounted for 75% or more of the cover. The Mixed Forest classification included forest cover in which neither deciduous nor evergreen species accounted for more than 75% of the cover. We restricted our selection of count locations in this way to increase the odds that species classified as forest-dependent were in fact breeding in forest. We analyzed landscapes of ten different radii surrounding point count locations: 0.2, 0.5, 1, 2, 4, 6, 8, 10, 12, and 16 km. Landscapes that overlapped with the Pennsylvania state border were omitted from analysis. Landscape sizes ranged over one order of magnitude at a high density in order to increase the likelihood of accurate identification of each species’ urbanization intensity scale of effect [53].

For each forest bird species, we selected the landscapes within the species’ range (Fig 1). To determine species ranges, we used PBBA block occurrence probabilities calculated using simple conditional autoregressive models accounting for the survey hours completed by each observer and land cover class proportions [54]. Block occurrence probabilities were assigned to the center of each block and then spatially interpolated for each species using kriging in ArcGIS to account for variation within survey blocks [55]. We classified land area with an interpolated occurrence probability ≥ 0.2 as lying within the species’ range. An occurrence probability of 0.2 can be interpreted as seeing the species in a given location once every five years on average–the duration of the PBBA.

### Urbanization intensity and other explanatory variables

We used the urbanization intensity variables and measures of landscape heterogeneity, habitat quality, and species detectability measured for a companion study [56]. For each landscape scale, urbanization intensity was quantified as the first and/or second components with eigenvalues > 1 [57] from a principal component analysis of six variables: the proportional area of landscapes in each of the four Developed classes of the NLCD, area-weighted average population density in landscapes, and area-weighted average housing density in landscapes. The first and second components, accounting for 62–76% of total variance, were selected at the 0.2 and 0.5 km scales while only the first component, accounting for 67–90% of total variance, was selected at larger scales. The first component was highly correlated with population and housing density (*r* > 0.90) and the areas of Developed land covers (*r* ≥ 0.70). The second component was highly correlated with High Intensity Developed cover (*r* ≥ 0.75).

We included six measures of landscape heterogeneity (forest amount, forest configuration, agriculture amount, mean elevation, elevation range, and land cover diversity), two measures of local habitat quality, and four measures of species detectability in the urbanization intensity analysis. Forest amount was the proportional area of landscapes in the NLCD Forest cover class, Deciduous, Mixed, or Evergreen, that best matched each forest species’ habitat affinity [52, 58]. For generalist forest species or those for which habitat affinity was unknown, the total of all Forest cover classes was used to estimate forest amount. Forest configuration was quantified for each species using the Forest class used to calculate forest amount for the species and the patch density and clumpiness index metrics [59]. Agriculture amount was the proportional area of landscapes in the NLCD Cultivated Crops and Pasture/Hay classes. The mean elevation and elevation range of landscapes were calculated using a 3.2-ft digital elevation model from the PAMAP Program [60]. We measured land cover diversity as Shannon’s diversity index of all NLCD land cover classes in landscapes. During point counts, PBBA staff recorded two indicators of local habitat quality: evidence of recent or active local land use change (presence or absence of change, such as logging or construction) and the identity of the dominant habitat type, e.g., coniferous forest, deciduous forest, or mixed forest, within 75m of the count location. Staff also recorded four metrics that could affect bird detectability: observer identity, survey start time, Julian date, and year. These variables represented variation in observer efficacy in counting birds, daily variation in the occurrence and vocalizations of birds due in part to weather conditions, and yearly variation in climatic conditions and species population sizes.

### Species traits

We collected data on the wingspan, body mass, dietary habits (frugivory, granivory, omnivory), foraging height, migratory status, nesting height, clutch size, number of fledglings per clutch, number of clutches per year, duration in the nest, cavity nesting, parental involvement in nest building, flock size, lifespan, song attributes, and territory size of each forest bird species (S2 File). We selected traits that were most commonly included, and found to be important, in previous studies of birds in urban environments. As such, our list does not include all previously studied traits such as habitat association. With the exception of song attributes, we gathered species trait data using the information in respected field guides or other sources (e.g. [58, 61]). We averaged values between sexes and across sources for continuous traits. If species could be classified into multiple categories, such as species with granivorous and omnivorous dietary habits, we assigned them to all applicable categories. Instead of classifying nesting species as ground, shrub, or tree nesters, we collected data on average nesting height in meters. We classified species as migratory or resident, with partially migratory species (species for which some but not all individuals migrate) falling into the latter category. We characterized the song attributes of each species by calculating the mean and range of song frequencies and song length from recordings for eastern and central North America [62] using the Praat software [63].

### Analyses

We tested for the influence of species traits on the magnitude and scale of effect of forest bird responses to urbanization intensity in two steps. First, we used logistic regressions and multi-model inference to estimate the association of urbanization intensity with the occurrence of each forest bird species, carrying out a separate analysis for each landscape scale. There was a degree of landscape overlap at all scales except the 200m scale. However, there is evidence that this overlap does not significantly impact results [64]. Although species occurrences do not imply population-level responses to urbanization intensity, we chose to use these data rather than abundances as our response variables because some species were too rare for meaningful analysis of their abundance data. By using occurrences, we maximized the number of species for which we could obtain urbanization intensity effect sizes and scales of effect and the sample size for the second step in our analyses. Models also included additional measures of landscape heterogeneity, local habitat quality, and species detectability. The absolute values of correlations between urbanization intensity and other explanatory variables were below the threshold of 0.70; above this value, collinearity may impair model fit [65]. We divided explanatory variables by their partial standard deviations–standard deviations divided by variable inflation factors, sample size, and number of predictor variables in models–so urbanization intensity effect sizes could be sensibly averaged across models [66]. We also standardized explanatory variables.

For each species and each landscape scale, we used Akaike's Information Criterion (AIC) to evaluate models that included every possible combination of explanatory variables. We calculated the full model-averaged effect size of the first component of urbanization intensity and its unconditional variance using models with ΔAIC ≤ 2 [67] for species and scales with unimodal effect sizes (97% of cases; [68]). The scale of effect of urbanization intensity for each species was the scale at which urbanization intensity had the largest absolute effect on species occurrence.

Second, we determined the effects of species traits on species’ urbanization intensity effect sizes and scales of effect using generalized linear models and multi-model inference. Species effect sizes, i.e., the model-averaged β coefficients for urbanization intensity at its scale of effect, were weighted by their inverse variance to account for the varying precision of estimates [68]. In order to determine whether species relatedness would violate model assumptions, we first performed univariate phylogenetic regressions of urbanization intensity effect sizes and scales of effect on each trait using randomly selected Ericson backbone trees from the online service BirdTree [69, 70]. No traits exhibited a phylogenetic signal (Pagel’s Lambda (λ)) significantly different from 0, indicating no influence of species relatedness on trait effects (S2 and S3 Tables).

Some species and traits were missing many values so we modeled the effects of traits in two groups: 1) traits with positive adjusted R^2^ values in univariate regressions (urbanization intensity effect size: clutch size, fledglings per clutch, frugivory, granivory, omnivory, cavity nesting, and migratory status (N = 30 species); urbanization intensity scale of effect: clutch size, duration in the nest, frugivory, granivory, omnivory, cavity nesting, migratory status, flock size, foraging height, lifespan, and wingspan (N = 26 species)), and 2) traits that had values for all species (body mass, clutch size, frugivory, granivory, omnivory, cavity nesting, and migratory status (N = 58 species)). With two exceptions (clutch size x fledglings per nest, and frugivory x omnivory in the urbanization intensity response analysis with the best-performing traits (|r| = 0.72 for both pairings)), the absolute values of correlations between pairs of traits in each analysis were low. Traits were transformed in the same manner as explanatory variables in logistic regression models by dividing trait values by their partial standard deviations and standardizing the resulting values.

We used AIC to evaluate models that included all possible combinations of traits for each group and response variable (urbanization intensity effect size and scale of effect) and calculated the full model-averaged effect size and its unconditional variance for each trait in best models. All averaged effect sizes were unimodal. We performed analyses in R, version 3.4.3 [71] using the ape [72], caper [73], fmsb [74], and MuMIn [75] packages.

## Results

### Forest bird responses to urbanization intensity

The average urbanization intensity effect size across species was -0.36 ± 0.49 (SE), with effect sizes for individual species ranging between -1.49 ± 0.50 and 0.74 ± 0.06 (Table 1). Of the 58 species analyzed, only 14 exhibited positive associations with urbanization intensity, whereas 44 species had negative associations. The average scale of effect of urbanization intensity across species was 4.87 ± 5.95 km (Table 1). Species exhibited scales of effect at each of the ten scales we examined, although half were most sensitive to urbanization intensity at scales ≤ 1 km.

**Table 1:** The magnitude and spatial scale of the effect of urbanization intensity on the occurrences of forest bird species in Pennsylvania (USA). Models of occurrence also accounted for other aspects of landscape heterogeneity, local habitat quality, and species detectability. Effect size was calculated as the model-averaged β coefficient of urbanization intensity at its scale of effect using models with ΔAIC ≤ 2. The sample size (N) for each species is the number of point locations surrounded by landscapes with radii equal to the scale of effect. Landscapes that intersected the state border were excluded from analyses, resulting in different numbers of landscapes at each scale.

Common name (s*cientific name*)	N	Effect size ± SE	Scale of effect (km)
Canada Warbler (*Cardellina canadensis*)	7010	-1.49 ± 0.50	1
Black-throated Blue Warbler (*Setophaga caerulescens*)	9222	-1.19 ± 0.21	1
Winter Wren (*Troglodytes hiemalis*)	5851	-1.16 ± 0.27	1
Louisiana Waterthrush (*Parkesia motacilla*)	13732	-1.12 ± 0.18	0.5
Least Flycatcher (*Empidonax minimus*)	10473	-1.09 ± 0.21	1
Hermit Thrush (*Catharus guttatus*)	11059	-1.06 ± 0.10	2
Pine Warbler (*Setophaga pinus*)	5849	-0.95 ± 0.31	16
Magnolia Warbler (*Setophaga magnolia*)	8610	-0.91 ± 0.13	1
Northern Parula (*Setophaga americana*)	8077	-0.90 ± 0.20	1
Yellow-bellied Sapsucker (*Sphyrapicus varius*)	6440	-0.87 ± 0.12	8
Mourning Warbler (*Geothlypis philadephia*)	2909	-0.86 ± 0.40	0.5
Worm-eating Warbler (*Helmitheros vermivorum*)	3047	-0.82 ± 0.32	0.2
Dark-eyed Junco (*Junco hyemalis*)	9295	-0.81 ± 0.10	8
Brown Creeper (*Certhia americana*)	3279	-0.78 ± 0.23	0.2
Blackburnian Warbler (*Setophaga fusca*)	9241	-0.72 ± 0.10	1
Golden-winged Warbler (*Vermivora chrysoptera*)	2010	-0.70 ± 0.32	2
Black-throated Green Warbler (*Setophaga virens*)	11842	-0.68 ± 0.07	1
Common Raven (*Corvus corax*)	13101	-0.66 ± 0.16	2
Ovenbird (*Seiurus aurocapilla*)	13701	-0.62 ± 0.05	16
Veery (*Catharus fuscescens*)	13071	-0.61 ± 0.06	0.2
Golden-crowned Kinglet (*Regulus satrapa*)	3701	-0.58 ± 0.31	2
Yellow-throated Vireo (*Vireo flavifrons*)	9641	-0.56 ± 0.14	6
Chestnut-sided Warbler (*Setophaga pensylvanica*)	13494	-0.54 ± 0.06	1
Hooded Warbler (*Setophaga citrina*)	6050	-0.54 ± 0.13	0.2
Swamp Sparrow (*Melospiza georgiana*)	7987	-0.52 ± 0.18	16
Great Crested Flycatcher (*Myiarchus crinitus*)	13748	-0.48 ± 0.10	16
Blue-headed Vireo (*Vireo solitarius*)	11593	-0.44 ± 0.08	1
Cerulean Warbler (*Setophaga cerulea*)	6782	-0.44 ± 0.14	6
Acadian Flycatcher (*Empidonax virescens*)	13761	-0.39 ± 0.06	0.2
Scarlet Tanager (*Piranga olivacea*)	7540	-0.34 ± 0.04	0.2
Blue-gray Gnatcatcher (*Polioptila caerulea*)	10547	-0.32 ± 0.08	0.5
Rose-breasted Grosbeak (*Pheucticus ludovicianus*)	11491	-0.31 ± 0.06	0.5
Yellow-billed Cuckoo (*Coccyzus americanus*)	16313	-0.29 ± 0.06	1
American Redstart (*Setophaga ruticilla*)	16208	-0.28 ± 0.04	0.2
Common Yellowthroat (*Geothlypis trichas*)	16407	-0.26 ± 0.03	0.2
Eastern Wood-Pewee (*Contopus virens*)	16407	-0.24 ± 0.03	0.2
Pileated Woodpecker (*Dryocopus pileatus*)	15378	-0.24 ± 0.07	6
Black-and-white Warbler (*Mniotilta varia*)	7204	-0.20 ± 0.07	0.2
Red-eyed Vireo (*Vireo olivaceus*)	11682	-0.19 ± 0.02	0.5
Hairy Woodpecker (*Picoides villosus*)	16407	-0.18 ± 0.07	0.2
Cedar Waxwing (*Bombycilla cedrorum*)	13776	-0.13 ± 0.06	16
Tree Swallow (*Tachycineta bicolor*)	16144	-0.12 ± 0.14	2
Ruby-throated Hummingbird (*Archilochus colubris*)	15391	-0.06 ± 0.06	6
Prairie Warbler (*Setophaga discolor*)	8658	-0.03 ± 0.08	0.2
Red-breasted Nuthatch (*Sitta canadensis*)	3743	0.01 ± 0.06	0.5
Purple Finch (*Haemorhous purpureus*)	10288	0.02 ± 0.05	10
Black-capped Chickadee (*Poecile atricapillus*)	14186	0.03 ± 0.03	10
White-breasted Nuthatch (*Sitta carolinensis*)	15231	0.03 ± 0.03	2
Indigo Bunting (*Passerina cyanea*)	15085	0.08 ± 0.02	8
Eastern Phoebe (*Sayornis phoebe*)	13776	0.09 ± 0.09	16
Yellow-throated Warbler (*Setophaga dominica*)	1150	0.13 ± 0.34	4
Chipping Sparrow (*Spizella passerina*)	16407	0.17 ± 0.02	0.2
Wood Thrush (*Hylocichla mustelina*)	14737	0.19 ± 0.04	10
American Crow (*Corvus brachyrhynchos*)	13776	0.22 ± 0.05	16
Tufted Titmouse (*Baeolophus bicolor*)	13776	0.25 ± 0.04	16
Northern Flicker (*Colaptes auratus*)	13776	0.45 ± 0.08	16
Kentucky Warbler (*Geothlypis formosa*)	3525	0.49 ± 0.47	12
Eastern Towhee (*Pipilo erythrophthalmus*)	13776	0.74 ± 0.06	16

N: number of landscapes analyzed at the species’ scale of effect. SE: standard error.

### Effects of species traits on urbanization intensity responses

**Fig 2:**
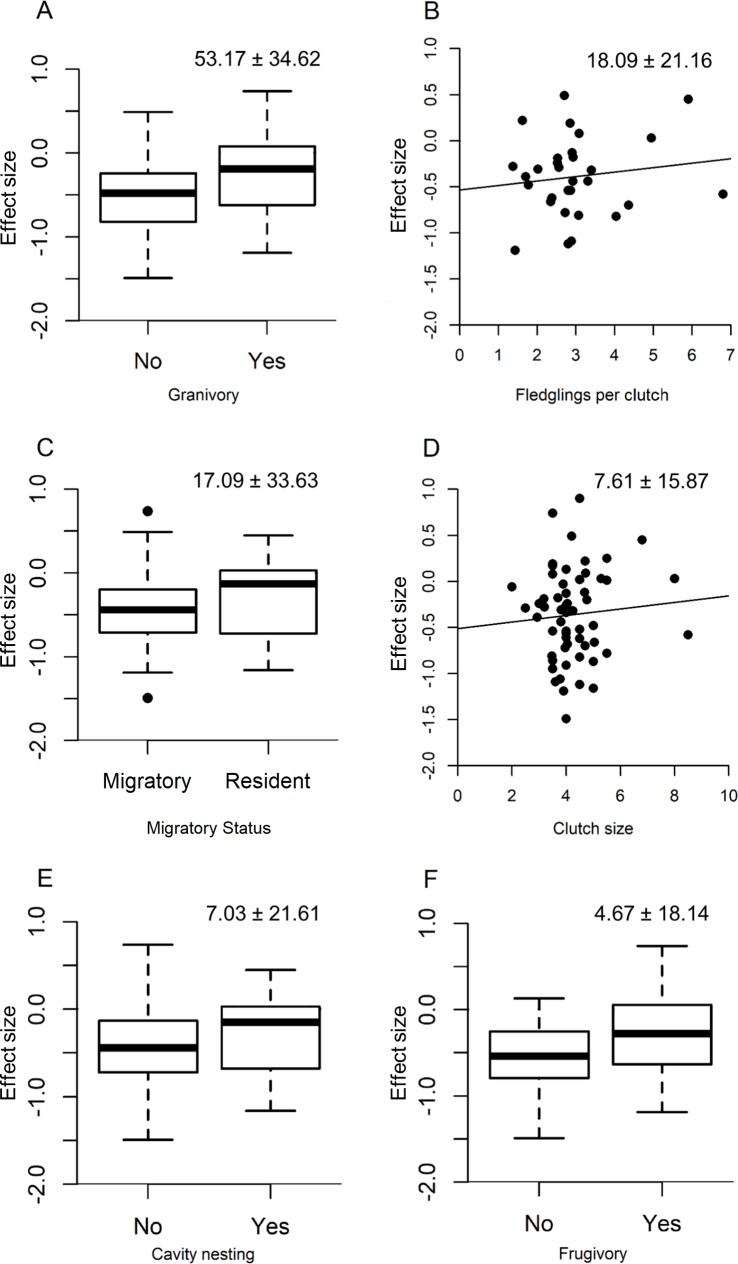
The effects of species traits on the magnitude of forest bird responses to urbanization intensity in Pennsylvania, USA. Traits are those in the best models among models that included only traits with positive adjusted R^2^ values in univariate regressions or models that included only traits with values for all species. The larger effect is shown for traits included in both sets of best models.

Clutch size, fledglings per clutch, frugivory, granivory, cavity nesting, and migratory status were included in the best models of the effects of species traits on the magnitude of species responses to urbanization intensity (Tables 2 and 3). All traits had a positive effect, with that of granivory being the largest, followed by fledglings per clutch, being a resident rather than a migratory species, clutch size, cavity nesting, and frugivory (Fig 2).

**Table 2:** The best models of the effects of species traits on the magnitude of forest bird responses to urbanization intensity in Pennsylvania, USA. Evaluated models included traits with positive adjusted R^2^ values in univariate regressions.

Model	K	AIC	ΔAIC	w_i_
Fledglings per clutch	3	362.15	0.00	0.30
Clutch size	3	362.53	0.38	0.25
Migratory status	3	363.03	0.88	0.19
Fledglings per clutch + migratory status	4	363.76	1.61	0.13
Fledglings per clutch + frugivory	4	363.82	1.67	0.13

K: number of estimated parameters (parameters include the coefficient of each explanatory variable, the model intercept, and the standard deviation of model error), AIC: Akaike’s Information Criterion, Δ_i_: AICi−minAIC for each model i, w_i_: Akaike weight, or probability of being the best model given the observed data and evaluated models.

**Table 3:** The best models of the effects of species traits on the magnitude of forest bird responses to urbanization intensity in Pennsylvania, USA. Evaluated models included traits with values for all species.

Model	K	AIC	ΔAIC	w_i_
Granivory	3	716.48	0.00	0.27
Cavity nesting + granivory	4	717.60	1.12	0.15
Clutch size + granivory	4	717.65	1.18	0.15
Granivory + migratory status	4	718.14	1.67	0.12
Migratory status	3	718.36	1.88	0.10
Frugivory + Granivory	4	718.36	1.89	0.10
Clutch size	3	718.39	1.91	0.10

K: number of estimated parameters (parameters include the coefficient of each explanatory variable, the model intercept, and the standard deviation of model error), AIC: Akaike’s Information Criterion, Δ_i_: AICi−minAIC for each model i, w_i_: Akaike weight, or probability of being the best model given the observed data and evaluated models

**Fig 3:**
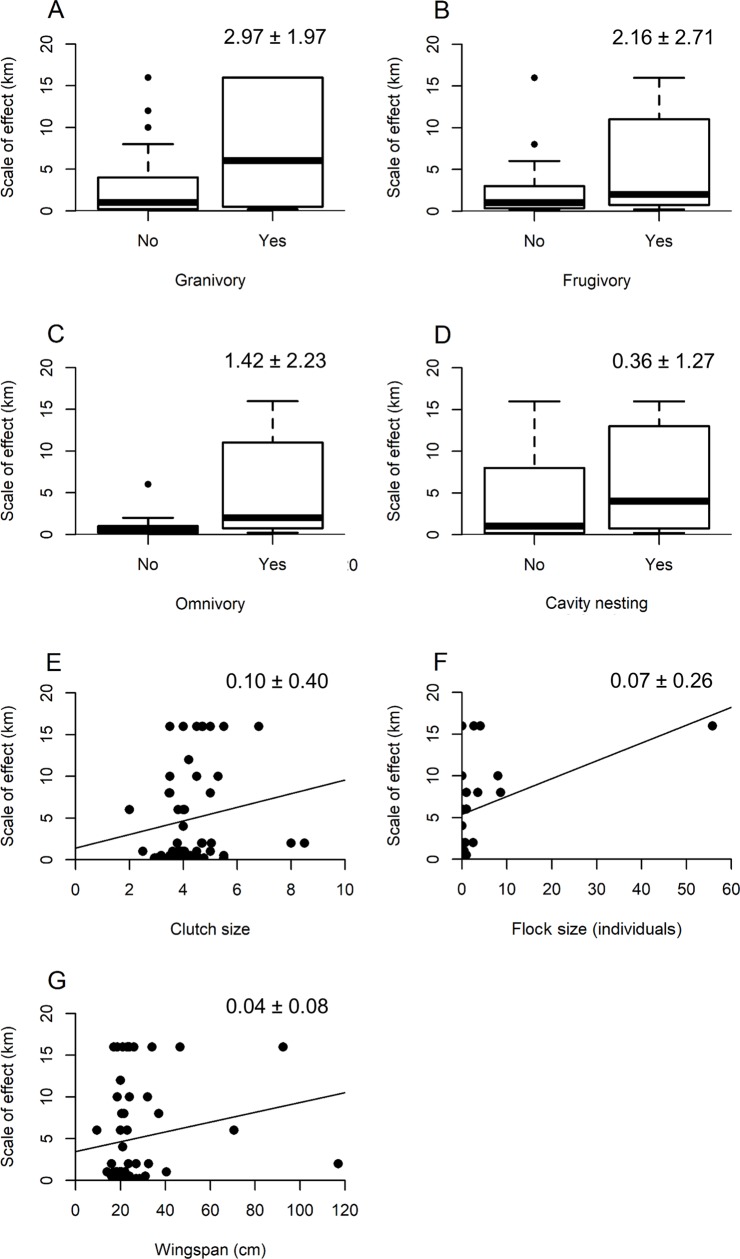
The effects of species traits on the spatial scale of forest bird responses to urbanization intensity in Pennsylvania, USA. Traits are those in the best models among models that included only traits with positive adjusted R^2^ values in univariate regressions or models that included only traits with values for all species. The larger effect is shown for traits included in both sets of best models.

Clutch size, frugivory, granivory, omnivory, cavity nesting, wingspan, and flock size were included in the best models of the effects of species traits on the spatial scale of species responses to urbanization intensity (Tables 4 and 5). All traits had a positive effect on the spatial scale at which species occurrences were most affected by urbanization intensity, with that of granivory being the largest, followed by frugivory, omnivory, cavity nesting, clutch size, flock size, and wingspan (Fig 3). There was no change to measured effect sizes if an apparent outlier (the Cedar Waxwing (*Bombycilla cedrorum*)) was excluded from the analysis.

**Table 4:** The best models of the effects of species traits on the scale of effect of forest bird responses to urbanization intensity in Pennsylvania, USA. Evaluated models included traits with positive adjusted R^2^ values in univariate regressions.

Model	K	AIC	ΔAIC	w_i_
Frugivory	3	168.33	0.00	0.13
Frugivory + granivory	4	168.82	0.49	0.10
Wingspan	3	168.91	0.58	0.10
Clutch size + frugivory	4	168.99	0.66	0.09
Granivory	3	169.23	0.90	0.08
Flock size + frugivory	4	169.41	1.08	0.07
Omnivory	3	169.41	1.08	0.07
Granivory + wingspan	4	169.48	1.15	0.07
Cavity nesting + frugivory	4	169.50	1.17	0.07
Cavity nesting	3	169.88	1.55	0.06
Frugivory + wingspan	4	170.17	1.84	0.05
Flock size + wingspan	4	170.28	1.95	0.05
Omnivory + wingspan	4	170.30	1.97	0.05

K: number of estimated parameters (parameters include the coefficient of each explanatory variable, the model intercept, and the standard deviation of model error), AIC: Akaike’s Information Criterion, Δ_i_: AICi−minAIC for each model i, w_i_: Akaike weight, or probability of being the best model given the observed data and evaluated models

**Table 5:** The best models of the effects of species traits on the scale of effect of forest bird responses to urbanization intensity in Pennsylvania, USA. Evaluated models included traits with values for all species.

Model	K	AIC	ΔAIC	w_i_
Frugivory + granivory	4	369.40	0.00	0.21
Granivory + omnivory	4	369.43	0.03	0.20
Granivory	3	369.48	0.08	0.20
Omnivory	3	370.04	0.64	0.15
Clutch size + frugivory + granivory	5	371.17	1.77	0.09
Cavity nesting + granivory	4	371.27	1.87	0.08
Frugivory + granivory + omnivory	5	371.38	1.98	0.08

K: number of estimated parameters (parameters include the coefficient of each explanatory variable, the model intercept, and the standard deviation of model error), AIC: Akaike’s Information Criterion, Δ_i_: AICi−minAIC for each model i, w_i_: Akaike weight, or probability of being the best model given the observed data and evaluated models

## Discussion

We show that resident forest bird species that are granivorous or frugivorous, cavity-nesting, and have larger clutch sizes and more fledglings per clutch are more likely to occur with increasing urbanization intensity in landscapes. In addition, the effect of urbanization intensity on forest birds manifested most strongly at larger spatial scales for granivorous, frugivorous, or omnivorous species that are cavity-nesting, have larger clutch sizes and longer wingspans, and flock in larger numbers. In the following, we discuss the possible mechanisms underlying these results and their implications for forest bird conservation in urbanizing landscapes.

In general, dietary traits had strong associations with both urbanization intensity effect size and scale of effect. Granivory had the largest positive effect on both the magnitude and spatial scale of forest bird responses to urbanization intensity. Urbanized landscapes–in our study, those with higher population and housing densities and covers of developed open space and low-, medium-, and high-intensity development–are typified by an abundance of seed-producing ornamental plants [76] as well as feeding tables and bird feeders [77]. Our results indicate that seed-eating forest bird species, such as the Black-capped Chickadee (*Poecile atricappilus*), are able to capitalize on these supplemental resources. In particular, several of the species that exhibited positive urbanization intensity effect sizes are known to make use of bird feeders and/or feeding tables, such as the White-breasted Nuthatch (*Sitta carolinensis*), at least intermittently. The frugivorous species we examined, such as the Purple Finch (*Haemorhous purpureus*) also appear to benefit from the proliferation of fruit-bearing shrubs and trees in urbanizing landscapes [78, 79]. The positive effect of granivory on the magnitude of forest bird responses to urbanization intensity has a corollary in studies of birds in general, in that particularly successful urban exploiters such as the Rock Pigeon (*Columba livia*) and the House Sparrow (*Passer domesticus*) are typically granivorous species [80]. The fact that granivorous forest species, as well as those with other dietary habitats, were most affected by urbanization intensity at larger spatial scales indicates that supplementary food resources in urban environments are not evenly distributed [80], resulting in species having to travel farther to obtain them. In addition, the positive association between omnivory and scale of effect indicates that the clumped distribution of resources in urban environments may not be restricted to supplementary foods but may be a general phenomenon characteristic of all food types, including prey.

We did not test for associations of insectivory with the magnitude and spatial scale of species responses to urbanization intensity because the species counted in the PBBA were in their nesting season, and would thus all be engaging in some degree of insectivory to feed hatchlings. Even so, species that are insectivorous in the non-breeding season may be characterized by traits in the breeding season that distinguish them from species with other dietary habits, such as nest shape and vulnerability to air pollution. The effects of these additional characteristics on urbanization intensity effect size and scale of effect remain to be investigated.

The reproductive traits of clutch size and fledglings per clutch were also relatively important determinants of the magnitude and spatial scale of forest bird responses to urbanization intensity in landscapes. Forest bird species that have higher reproductive output, such as the Northern Flicker (*Colaptes auratus*), occurred more frequently with increasing urbanization intensity. The additional eggs and fledglings of such species may buffer the effects of losses to predation and parasitism–the latter by the Brown-headed Cowbird (*Molothrus ater*), which is positively associated with suburbanization [81, 82] and the sole avian nest parasite in our study area–suffered by less prolific species. Species with higher reproductive output have also been found to be less vulnerable to the negative effects of roads [83]. In addition, forest bird species with larger clutch sizes were most strongly affected by urbanization intensity at larger spatial scales. We speculate that species that produce larger clutches must move over greater distances in urbanizing landscapes to obtain sufficient resources for their young. This hypothesis remains to be tested.

The positive effect of being a resident rather than a migratory species on the magnitude of forest bird responses to urbanization intensity was similar in size to that of fledglings per clutch. Being a resident species is also known to be associated with urbanization success in birds in general [20, 30, 42], and is represented in our sample by species such as the American Crow (*Corvus brachyrhynchos*) and the White-breasted Nuthatch (*S*. *carolinensis*). Possible explanations for the positive effect of being a resident species include resident species occupying high-quality nesting habitat in urbanizing landscapes before migratory species arrive [20, 38]; resident species having a more comprehensive knowledge of the distribution of urban resources, and thereby garnering a larger share of them, than migratory species [20]; resident species preferentially benefiting from the increasing occurrence of winter bird-feeding in urbanizing landscapes [84]; and, the lack of migratory mortality among resident species [41], although the latter may be offset by higher winter mortality in our study area.

Forest bird species in Pennsylvania that nest in cavities, such as the Tufted Titmouse (*Baeolophus bicolor*), were more positively associated with increasing urbanization intensity and exhibited a larger scale of effect of urbanization intensity than non-cavity-nesters. Recent research in Europe has also found that cavity-nesters, including threatened species, do relatively well in urban settings [85]. The positive effect of cavity-nesting on urbanization intensity effect size may not be due to an increase in the occurrence of natural cavities in trees, which typically declines in urbanizing landscapes [86]. Instead, cavity-nesters may be less vulnerable to urban-associated disturbances such as increased predation, although support for this hypothesis is equivocal [38, 85, 87, 88]. Alternatively, the positive effect of cavity-nesting on urbanization intensity effect size may be explained by the fairly large proportion of cavity-nesting species in our analysis that nest in nest boxes and other human-made structures, such as the Black-Capped Chickadee (*P*. *atricapillus*). While we did not assess whether there was a difference in urbanization intensity effect size between primary and secondary cavity nesters, it is possible that this distinction may be impactful in that the occurrence of secondary nesters would be more positively affected by urbanization intensity than that of primary nesters, although further research is needed [89]. As with the distribution of supplementary food resources, the availability of cavity-bearing trees in urbanizing landscapes appears to be unevenly distributed, resulting in cavity-nesting forest bird species being most strongly affected by urbanization intensity at larger spatial scales. The uneven distribution of cavity-bearing trees may be particularly pronounced in urbanizing landscapes if such trees are restricted to spatially disjunct nature reserves [90].

Finally, flock size and wingspan had relatively small positive effects on the spatial scale at which urbanization intensity most strongly affected forest bird species. Species with larger flock sizes, such as the Cedar Waxwing (*B*. *cedrorum)* need larger landscapes to adequately feed all members of the flock [91] and thus would exhibit larger scales of effect of urbanization intensity. Similarly, bird species with longer wingspans, such as the Common Raven (*Corvus corax*) disperse over longer distances [92] over which they would be most exposed to the effects of urbanization intensity. Alternatively, the positive association between wingspan and urbanization intensity scale of effect may be driven by the spatial clustering of food resources in urban environments, which longer-winged species would be better able to take advantage of.

Our results point to several management strategies that may improve the conservation of forest bird species in urbanizing landscapes should the underlying mechanisms that we propose be confirmed by future research. The positive effects of dietary habit, particularly granivory, being a resident species, and cavity nesting on the magnitude of forest bird responses to urbanization intensity imply that public and private land managers in urbanizing landscapes maintain or increase the year-round availability of food and nesting resources for forest birds. Municipal urban forest management plans that emphasize the safe preservation and creation of cavity-bearing trees and public outreach and educational efforts focused on winter bird-feeding, nest box availability, and backyard wildlife habitat certification are ways in which this goal may be accomplished. If feasible, these initiatives should be implemented in a manner that increases the spatial dispersion of resources across landscapes, thereby shortening the distances over which granivorous, frugivorous, omnivorous, and cavity-nesting forest birds must move and consequently reducing their probability of movement mortality.

Although important to maintain or increase the populations of some forest bird species, increasing the availability and accessibility of food and nesting resources only targets species that are already doing relatively well in urbanizing landscapes and in our study, this was a minority: only 14 of 44 species, or 32%, had positive urbanization intensity effect sizes. Urban land managers should consider prioritizing strategies that benefit forest bird species that are more negatively affected by urbanization intensity, i.e., migratory species with small clutches and few fledglings per clutch. Specifically, we suggest that public land managers and private landowners limit the disproportionally negative impact of nest predation on forest bird species with low reproductive productivity by restricting the activity of domestic cats [93] and corvids [94] in urbanizing landscapes and employ strategies, such as reduced night lighting, that minimize bird collisions with buildings, which may disproportionally affect migratory species [95]. The cultivation of mature vegetation composed of native tree and shrub species may also favor migratory species if used as stopover habitat. Finally, we note that none of the management strategies that we propose here have been empirically tested. This remains an important frontier in urban forest bird species conservation.

In conclusion, our results indicate that the resident forest bird species that are most positively associated with urbanization intensity are those that are granivorous or frugivorous, have larger clutches and more fledglings per clutch, and are cavity-nesting. In addition, urbanization intensity appears to affect granivorous, frugivorous, or omnivorous species, cavity-nesters, species with larger clutch sizes or longer wings, and species that flock in greater numbers most strongly at larger spatial scales. Based on their trait characteristics, the forest bird species in our study expected to have the strongest positive association with urbanization intensity is the Northern Flicker (*C*. *auratus)*, while the species expected to have the most negative association is the Ruby-throated Hummingbird (*A*. *colubris*). And, based on their trait characteristics, the forest bird species in our study expected to be influenced by urbanization intensity at the largest scale is the Common Raven (*C*. *corax*), while the species expected to be influenced by urbanization at the smallest scale is the Golden-winged Warbler (*Vermivora chrysoptera*). To our knowledge, our study represents the first direct tests of the effects of species traits on the magnitude and spatial scale of the effect of urbanization on forest birds, as well as the first evidence that migratory status, clutch size, wingspan, and fledglings per clutch are important determinants of the responses of forest birds to urbanization. Future research should focus on testing the mechanisms we propose to explain the effects of species traits and the effectiveness of the management strategies they imply.

## Supporting information

S1 Fig (DOCX)

S1 File Methods used to classify forest-dependent bird species. (DOCX)

S2 File Forest bird species trait values, codes, and sources. (XLSX)

S3 File Results of univariate phylogenetic regressions of urbanization intensity effect sizes and scales of effect on forest bird species traits. (DOCX)

S1 Table (DOCX)

S2 Table Results from general linear models of the effects of traits on forest bird species’ responses to urbanization intensity in Pennsylvania, USA. (DOCX)

S3 Table Results from general linear models of the effects of traits on scales of effect of forest bird species’ responses to urbanization intensity in Pennsylvania, USA. (DOCX)
