# Supplementary material for: The traits that predict the magnitude and spatial scale of forest bird responses to urbanization intensity
Source: PLoS One. 2019 Jul 25;14(7):e0220120. doi: 10.1371/journal.pone.0220120 (PMC6657869; doi:10.1371/journal.pone.0220120)
Supplement: S1 Fig — (DOCX) [file pone.0220120.s001.docx]

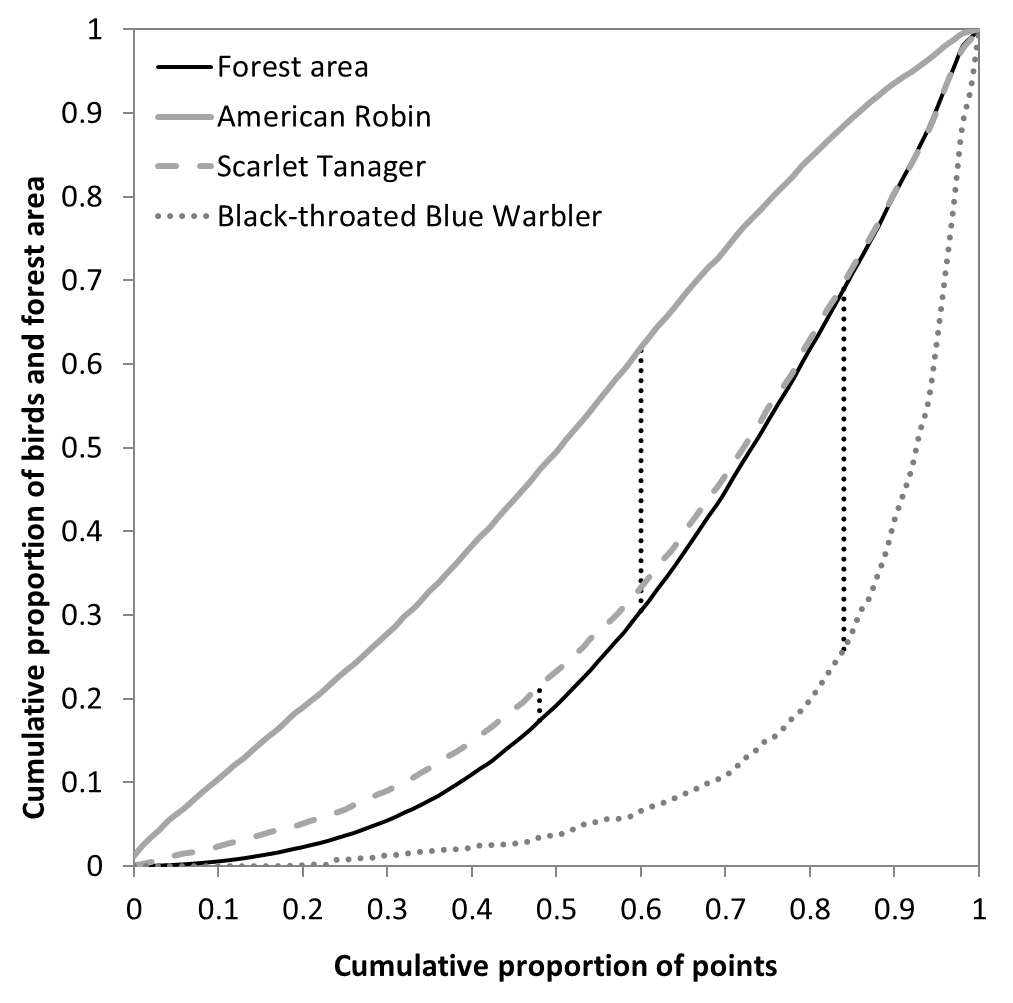


**S1 Fig.** Example of classification of species as "forest-dependent" based on the cumulative distribution of their counts and the cumulative distribution of forest cover in landscapes surrounding count locations (N = 33,763). This example is for landscapes of 1-km radius. Dotted black lines represent the largest deviances between the bird count curve and the forest cover curve, as measured by D, the Kolmogorov-Smirnov (KS) test statistic. The null hypothesis in these tests is that the bird count and forest cover distributions are identical. In other words, the cumulative increase in bird counts in landscapes occurs at the same rate as the cumulative increase in forest cover in landscapes. If this is the case, then the species in question increased in abundance in proportion to the amount of forest in landscapes.

The Black-throated Blue Warbler's (*Setophaga caerulescens*) count distribution differed significantly from that of forest cover (*D* = 0.43, *p* < 0.001). In this case, the top 16% most forested landscapes encompassed 31% of all forest cover but accounted for 74% of birds, indicating that this species preferred landscapes with large amounts of forest. The Scarlet Tanager's (*Piranga olivacea*) count distribution did not differ significantly from that of forest cover (*D* = 0.04, *p* = 0.851), indicating that this species occurred in proportion to forest cover in landscapes. In other words, the Scarlet Tanager required forest in landscapes but occurred across a broad range of forest amounts. The American Robin's (*Turdus migratorius*) count distribution also differed significantly from that of forest cover (*D* = -0.31, *p* < 0.001) but a large proportion of American Robins occurred in landscapes with less forest, indicating that this species did not depend on forest habitat in landscapes.

Species were classified as "forest-dependent" or "forest-independent" based on the results of KS tests at 10 landscape scales (Table A). In the cases of the above three species, the Black-throated Blue Warbler's results at all 10 scales indicated that it preferred landscapes with large amounts of forest, the Scarlet Tanager was found in proportion to forest cover at all 10 scales, and the American Robin was found disproportionately in less forested landscapes at all 10 scales. The Black-throated Blue Warbler and Scarlet Tanager were therefore classified as forest-dependent species and the American Robin was classified as a forest-independent species. In general, species were classified as forest-dependent or forest-independent based on whether the majority of their test statistic values across scales were significantly positive or significantly negative, respectively. Species that did not meet either of these criteria were also classified as forest-dependent.
