# Supplementary material for: The traits that predict the magnitude and spatial scale of forest bird responses to urbanization intensity
Source: PLoS One. 2019 Jul 25;14(7):e0220120. doi: 10.1371/journal.pone.0220120 (PMC6657869; doi:10.1371/journal.pone.0220120)
Supplement: S1 File — (DOCX) [file pone.0220120.s002.docx]

**S1 File.** Methods used to classify forest-dependent bird species.


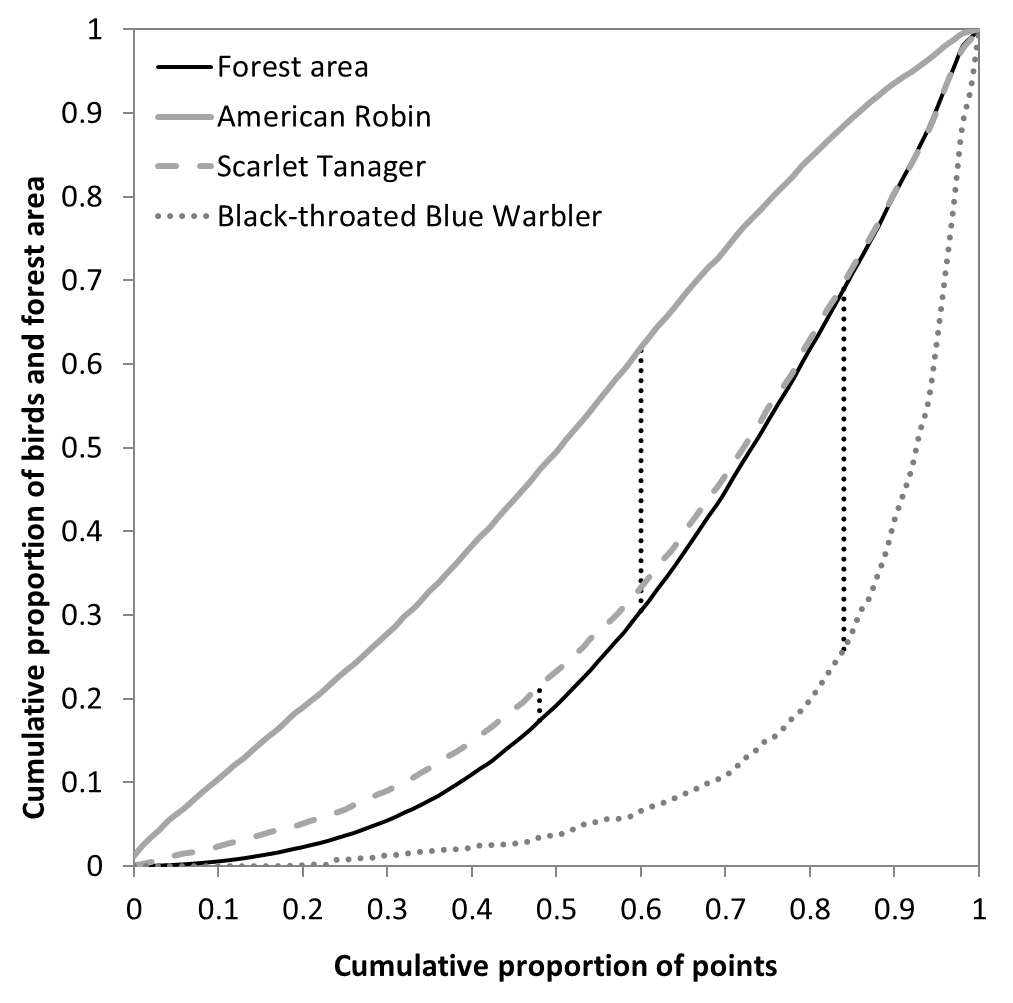


**S1 Fig.** Example of classification of species as "forest-dependent" based on the cumulative distribution of their counts and the cumulative distribution of forest cover in landscapes surrounding count locations (N = 33,763). This example is for landscapes of 1-km radius. Dotted black lines represent the largest deviances between the bird count curve and the forest cover curve, as measured by D, the Kolmogorov-Smirnov (KS) test statistic. The null hypothesis in these tests is that the bird count and forest cover distributions are identical. In other words, the cumulative increase in bird counts in landscapes occurs at the same rate as the cumulative increase in forest cover in landscapes. If this is the case, then the species in question increased in abundance in proportion to the amount of forest in landscapes.

The Black-throated Blue Warbler's (*Setophaga caerulescens*) count distribution differed significantly from that of forest cover (*D* = 0.43, *p* < 0.001). In this case, the top 16% most forested landscapes encompassed 31% of all forest cover but accounted for 74% of birds, indicating that this species preferred landscapes with large amounts of forest. The Scarlet Tanager's (*Piranga olivacea*) count distribution did not differ significantly from that of forest cover (*D* = 0.04, *p* = 0.851), indicating that this species occurred in proportion to forest cover in landscapes. In other words, the Scarlet Tanager required forest in landscapes but occurred across a broad range of forest amounts. The American Robin's (*Turdus migratorius*) count distribution also differed significantly from that of forest cover (*D* = -0.31, *p* < 0.001) but a large proportion of American Robins occurred in landscapes with less forest, indicating that this species did not depend on forest habitat in landscapes.

Species were classified as "forest-dependent" or "forest-independent" based on the results of KS tests at 10 landscape scales (Table A). In the cases of the above three species, the Black-throated Blue Warbler's results at all 10 scales indicated that it preferred landscapes with large amounts of forest, the Scarlet Tanager was found in proportion to forest cover at all 10 scales, and the American Robin was found disproportionately in less forested landscapes at all 10 scales. The Black-throated Blue Warbler and Scarlet Tanager were therefore classified as forest-dependent species and the American Robin was classified as a forest-independent species. In general, species were classified as forest-dependent or forest-independent based on whether the majority of their test statistic values across scales were significantly positive or significantly negative, respectively. Species that did not meet either of these criteria were also classified as forest-dependent.

**S1 Table.** Kolmogorov-Smirnov test statistic values for each of 101 bird species and 10 landscapes scales. Dark gray shading: *p* < 0.01; light gray shading: *p* < 0.05; no shading: *p* ≥ 0.05. Significant (*p* < 0.05) positive values indicate a large proportion of the individuals of a species occurred in the landscapes with the most forest, significant negative values indicate a large proportion of the individuals of a species occurred in the landscapes with the least forest, and non-significant values indicate a species occurred in proportion to forest cover. Species were classified as forest-dependent or forest-independent based on whether the majority of their test statistic values across scales were significantly positive or significantly negative, respectively. Species that did not meet either of these criteria were also classified as forest-dependent.

| Guild | Species | Landscape scale (km) | | | | | | | | | |
| --- | --- | --- | --- | --- | --- | --- | --- | --- | --- | --- | --- |
|  |  | 0.2 | 0.5 | 1 | 2 | 4 | 6 | 8 | 10 | 12 | 16 |
| Forest-dependent | Black-throated Blue Warbler | 0.35 | 0.40 | 0.43 | 0.47 | 0.49 | 0.51 | 0.52 | 0.51 | 0.49 | 0.49 |
|  | (*Setophaga caerulescens*) |  |  |  |  |  |  |  |  |  |  |
| Forest-dependent | Canada Warbler | 0.32 | 0.41 | 0.39 | 0.40 | 0.43 | 0.45 | 0.46 | 0.42 | 0.40 | 0.37 |
|  | (*Cardellina canadensis*) |  |  |  |  |  |  |  |  |  |  |
| Forest-dependent | Winter Wren | 0.20 | 0.20 | 0.28 | 0.36 | 0.41 | 0.43 | 0.43 | 0.44 | 0.44 | 0.47 |
|  | (*Troglodytes hiemalis*) |  |  |  |  |  |  |  |  |  |  |
| Forest-dependent | Brown Creeper | 0.22 | 0.24 | 0.29 | 0.33 | 0.36 | 0.38 | 0.38 | 0.38 | 0.37 | 0.37 |
|  | (*Certhia americana*) |  |  |  |  |  |  |  |  |  |  |
| Forest-dependent | Blackburnian Warbler | 0.18 | 0.22 | 0.29 | 0.35 | 0.39 | 0.41 | 0.43 | 0.44 | 0.44 | 0.43 |
|  | (*Setophaga fusca*) |  |  |  |  |  |  |  |  |  |  |
| Forest-dependent | Magnolia Warbler | 0.18 | 0.21 | 0.25 | 0.31 | 0.36 | 0.38 | 0.39 | 0.40 | 0.40 | 0.41 |
|  | (*Setophaga magnolia*) |  |  |  |  |  |  |  |  |  |  |
| Forest-dependent | Golden-crowned Kinglet | 0.11 | 0.20 | 0.24 | 0.26 | 0.31 | 0.36 | 0.38 | 0.39 | 0.41 | 0.43 |
|  | (*Regulus satrapa*) |  |  |  |  |  |  |  |  |  |  |
| Forest-dependent | Blue-headed Vireo | 0.15 | 0.19 | 0.25 | 0.29 | 0.33 | 0.35 | 0.36 | 0.37 | 0.38 | 0.37 |
|  | (*Vireo solitarius*) |  |  |  |  |  |  |  |  |  |  |
| Forest-dependent | Hermit Thrush | 0.19 | 0.17 | 0.21 | 0.26 | 0.31 | 0.34 | 0.35 | 0.35 | 0.36 | 0.37 |
|  | (*Catharus guttatus*) |  |  |  |  |  |  |  |  |  |  |
| Forest-dependent | Dark-eyed Junco | 0.18 | 0.18 | 0.24 | 0.28 | 0.31 | 0.33 | 0.34 | 0.35 | 0.34 | 0.35 |
|  | (*Junco hyemalis*) |  |  |  |  |  |  |  |  |  |  |
| Forest-dependent | Black-throated Green Warbler | 0.14 | 0.19 | 0.21 | 0.25 | 0.28 | 0.31 | 0.31 | 0.32 | 0.32 | 0.32 |
|  | (*Setophaga virens*) |  |  |  |  |  |  |  |  |  |  |
| Forest-dependent | Red-breasted Nuthatch | 0.08 | 0.11 | 0.17 | 0.22 | 0.30 | 0.29 | 0.33 | 0.33 | 0.34 | 0.35 |
|  | (*Sitta canadensis*) |  |  |  |  |  |  |  |  |  |  |
| Forest-dependent | Black-and-white Warbler | -0.08 | 0.09 | 0.15 | 0.21 | 0.24 | 0.25 | 0.27 | 0.26 | 0.27 | 0.25 |
|  | (*Mniotilta varia*) |  |  |  |  |  |  |  |  |  |  |
| Forest-dependent | Common Raven | -0.13 | 0.11 | 0.13 | 0.19 | 0.22 | 0.24 | 0.25 | 0.26 | 0.27 | 0.29 |
|  | (*Corvus corax*) |  |  |  |  |  |  |  |  |  |  |
| Forest-dependent | Yellow-rumped Warbler | 0.07 | 0.09 | 0.16 | 0.19 | 0.25 | 0.27 | 0.28 | 0.29 | 0.29 | 0.31 |
|  | (*Setophaga coronata*) |  |  |  |  |  |  |  |  |  |  |
| Forest-dependent | Yellow-bellied Sapsucker | 0.15 | 0.11 | 0.14 | 0.18 | 0.22 | 0.25 | 0.27 | 0.28 | 0.29 | 0.31 |
|  | (*Sphyrapicus varius*) |  |  |  |  |  |  |  |  |  |  |
| Forest-dependent | Golden-winged Warbler | -0.25 | -0.12 | -0.09 | 0.13 | 0.19 | 0.21 | 0.24 | 0.27 | 0.31 | 0.31 |
|  | (*Vermivora chrysoptera*) |  |  |  |  |  |  |  |  |  |  |
| Forest-dependent | Least Flycatcher | -0.14 | 0.10 | 0.12 | 0.15 | 0.19 | 0.21 | 0.21 | 0.21 | 0.22 | 0.22 |
|  | (*Empidonax minimus*) |  |  |  |  |  |  |  |  |  |  |
| Forest-dependent | Mourning Warbler | -0.19 | -0.07 | 0.10 | 0.14 | 0.17 | 0.20 | 0.22 | 0.23 | 0.25 | 0.26 |
|  | (*Geothlypis philadelphia*) |  |  |  |  |  |  |  |  |  |  |
| Forest-dependent | Pine Warbler | -0.13 | -0.07 | 0.09 | 0.12 | 0.19 | 0.21 | 0.22 | 0.20 | 0.20 | 0.22 |
|  | (*Setophaga pinus*) |  |  |  |  |  |  |  |  |  |  |
| Forest-dependent | Northern Parula | -0.22 | -0.12 | -0.06 | 0.08 | 0.13 | 0.15 | 0.17 | 0.17 | 0.16 | 0.16 |
|  | (*Setophaga americana*) |  |  |  |  |  |  |  |  |  |  |
| Forest-dependent | Veery | -0.12 | 0.05 | 0.08 | 0.10 | 0.13 | 0.15 | 0.15 | 0.15 | 0.15 | 0.15 |
|  | (*Catharus fuscescens*) |  |  |  |  |  |  |  |  |  |  |
| Forest-dependent | Cerulean Warbler | -0.13 | -0.06 | 0.07 | 0.11 | 0.16 | 0.16 | 0.17 | 0.15 | 0.15 | 0.13 |
|  | (*Setophaga cerulea*) |  |  |  |  |  |  |  |  |  |  |
| Forest-dependent | Chestnut-sided Warbler | -0.12 | -0.03 | 0.05 | 0.08 | 0.11 | 0.12 | 0.13 | 0.14 | 0.15 | 0.16 |
|  | (*Setophaga pensylvanica*) |  |  |  |  |  |  |  |  |  |  |
| Forest-dependent | Ovenbird | -0.13 | -0.06 | 0.02 | 0.05 | 0.08 | 0.09 | 0.10 | 0.10 | 0.10 | 0.10 |
|  | (*Seiurus aurocapillus*) |  |  |  |  |  |  |  |  |  |  |
| Forest-dependent | American Redstart | -0.15 | -0.08 | -0.03 | 0.04 | 0.06 | 0.07 | 0.08 | 0.09 | 0.08 | 0.08 |
|  | (*Setophaga ruticilla*) |  |  |  |  |  |  |  |  |  |  |
| Forest-dependent | Red-eyed Vireo | -0.17 | -0.08 | -0.04 | -0.01 | 0.04 | 0.05 | 0.06 | 0.06 | 0.07 | 0.07 |
|  | (*Vireo olivaceus*) |  |  |  |  |  |  |  |  |  |  |
| Forest-dependent | Scarlet Tanager | -0.16 | -0.08 | -0.04 | -0.01 | 0.04 | 0.05 | 0.05 | 0.05 | 0.05 | 0.05 |
|  | (*Piranga olivacea*) |  |  |  |  |  |  |  |  |  |  |
| Forest-dependent | Hooded Warbler | -0.15 | -0.08 | -0.04 | -0.02 | 0.03 | 0.04 | 0.04 | 0.04 | 0.04 | 0.04 |
|  | (*Setophaga citrina*) |  |  |  |  |  |  |  |  |  |  |
| Forest-dependent | Louisiana Waterthrush | -0.19 | -0.10 | -0.06 | -0.03 | 0.07 | 0.07 | 0.08 | 0.07 | -0.07 | -0.08 |
|  | (*Parkesia motacilla*) |  |  |  |  |  |  |  |  |  |  |
| Forest-dependent | Acadian Flycatcher | -0.16 | -0.08 | -0.04 | -0.03 | -0.02 | -0.03 | -0.04 | -0.05 | -0.05 | -0.07 |
|  | (*Empidonax virescens*) |  |  |  |  |  |  |  |  |  |  |
| Forest-dependent | Pileated Woodpecker | -0.19 | -0.11 | -0.07 | -0.05 | -0.04 | -0.04 | -0.03 | -0.03 | -0.04 | -0.05 |
|  | (*Dryocopus pileatus*) |  |  |  |  |  |  |  |  |  |  |
| Forest-dependent | Black-billed Cuckoo | -0.23 | -0.15 | -0.10 | -0.06 | -0.06 | 0.06 | 0.08 | 0.08 | 0.08 | 0.08 |
|  | (*Coccyzus erythropthalmus*) |  |  |  |  |  |  |  |  |  |  |
| Forest-dependent | Black-capped Chickadee | -0.21 | -0.12 | -0.07 | -0.05 | -0.02 | 0.03 | 0.04 | 0.04 | 0.05 | 0.05 |
|  | (*Poecile atricapillus*) |  |  |  |  |  |  |  |  |  |  |
| Forest-dependent | Ruby-throated Hummingbird | -0.22 | -0.14 | -0.09 | -0.06 | -0.03 | -0.03 | 0.01 | -0.01 | -0.01 | -0.01 |
|  | (*Archilochus colubris*) |  |  |  |  |  |  |  |  |  |  |
| Forest-dependent | Hairy Woodpecker | -0.23 | -0.15 | -0.12 | -0.09 | -0.05 | -0.04 | -0.02 | -0.02 | -0.02 | 0.02 |
|  | (*Picoides villosus*) |  |  |  |  |  |  |  |  |  |  |
| Forest-dependent | Rose-breasted Grosbeak | -0.22 | -0.14 | -0.09 | -0.08 | -0.06 | -0.05 | -0.05 | -0.05 | -0.04 | 0.04 |
|  | (*Pheucticus ludovicianus*) |  |  |  |  |  |  |  |  |  |  |
| Forest-dependent | Eastern Towhee | -0.21 | -0.13 | -0.09 | -0.08 | -0.06 | -0.06 | -0.06 | -0.05 | -0.06 | -0.06 |
|  | (*Pipilo erythrophthalmus*) |  |  |  |  |  |  |  |  |  |  |
| Forest-dependent | White-breasted Nuthatch | -0.23 | -0.15 | -0.11 | -0.08 | -0.07 | -0.06 | -0.06 | -0.04 | -0.04 | -0.04 |
|  | (*Sitta carolinensis*) |  |  |  |  |  |  |  |  |  |  |
| Forest-dependent | Yellow-billed Cuckoo | -0.24 | -0.16 | -0.11 | -0.08 | -0.06 | -0.05 | -0.05 | -0.05 | -0.05 | -0.05 |
|  | (*Coccyzus americanus*) |  |  |  |  |  |  |  |  |  |  |
| Forest-dependent | Worm-eating Warbler | -0.24 | -0.16 | -0.12 | -0.07 | -0.05 | -0.06 | -0.07 | -0.08 | -0.08 | -0.08 |
|  | (*Helmitheros vermivorum*) |  |  |  |  |  |  |  |  |  |  |
| Forest-dependent | Blue-gray Gnatcatcher | -0.24 | -0.18 | -0.14 | -0.10 | -0.09 | -0.08 | -0.08 | -0.08 | -0.08 | -0.08 |
|  | (*Polioptila caerulea*) |  |  |  |  |  |  |  |  |  |  |
| Forest-dependent | Yellow-throated Vireo | -0.26 | -0.18 | -0.15 | -0.14 | -0.14 | -0.15 | -0.14 | -0.13 | -0.13 | -0.13 |
|  | (*Vireo flavifrons*) |  |  |  |  |  |  |  |  |  |  |
| Forest-dependent | Prairie Warbler | -0.26 | -0.18 | -0.15 | -0.13 | -0.15 | -0.15 | -0.14 | -0.15 | -0.14 | -0.14 |
|  | (*Setophaga discolor*) |  |  |  |  |  |  |  |  |  |  |
| Forest-dependent | Purple Finch | -0.32 | -0.19 | -0.12 | -0.08 | -0.04 | -0.05 | -0.04 | 0.04 | 0.06 | 0.07 |
|  | (*Haemorhous purpureus*) |  |  |  |  |  |  |  |  |  |  |
| Forest-dependent | Common Yellowthroat | -0.27 | -0.19 | -0.16 | -0.14 | -0.12 | -0.11 | -0.10 | -0.09 | -0.09 | -0.08 |
|  | (*Geothlypis trichas*) |  |  |  |  |  |  |  |  |  |  |
| Forest-dependent | Indigo Bunting | -0.30 | -0.22 | -0.18 | -0.16 | -0.13 | -0.11 | -0.10 | -0.10 | -0.09 | -0.08 |
|  | (*Passerina cyanea*) |  |  |  |  |  |  |  |  |  |  |
| Forest-dependent | Eastern Phoebe | -0.29 | -0.20 | -0.17 | -0.15 | -0.13 | -0.13 | -0.12 | -0.11 | -0.11 | -0.09 |
|  | (*Sayornis phoebe*) |  |  |  |  |  |  |  |  |  |  |
| Forest-dependent | Cedar Waxwing | -0.30 | -0.23 | -0.19 | -0.16 | -0.13 | -0.13 | -0.12 | -0.11 | -0.11 | -0.09 |
|  | (*Bombycilla cedrorum*) |  |  |  |  |  |  |  |  |  |  |
| Forest-dependent | Kentucky Warbler | -0.26 | -0.19 | -0.16 | -0.17 | -0.15 | -0.15 | -0.13 | -0.13 | -0.14 | -0.13 |
|  | (*Geothlypis formosa*) |  |  |  |  |  |  |  |  |  |  |
| Forest-dependent | Eastern Wood-Pewee | -0.28 | -0.21 | -0.19 | -0.16 | -0.15 | -0.14 | -0.15 | -0.15 | -0.15 | -0.17 |
|  | (*Contopus virens*) |  |  |  |  |  |  |  |  |  |  |
| Forest-dependent | American Crow | -0.31 | -0.24 | -0.20 | -0.18 | -0.16 | -0.15 | -0.15 | -0.14 | -0.13 | -0.12 |
|  | (*Corvus brachyrhynchos*) |  |  |  |  |  |  |  |  |  |  |
| Forest-dependent | Yellow-throated Warbler | -0.26 | -0.23 | -0.20 | -0.17 | -0.19 | -0.16 | -0.15 | -0.14 | -0.16 | -0.15 |
|  | (*Setophaga dominica*) |  |  |  |  |  |  |  |  |  |  |
| Forest-dependent | Chipping Sparrow | -0.33 | -0.25 | -0.22 | -0.19 | -0.17 | -0.16 | -0.15 | -0.14 | -0.13 | -0.13 |
|  | (*Spizella passerina*) |  |  |  |  |  |  |  |  |  |  |
| Forest-dependent | Tufted Titmouse | -0.30 | -0.24 | -0.21 | -0.19 | -0.17 | -0.17 | -0.17 | -0.16 | -0.16 | -0.16 |
|  | (*Baeolophus bicolor*) |  |  |  |  |  |  |  |  |  |  |
| Forest-dependent | Wood Thrush | -0.29 | -0.23 | -0.20 | -0.19 | -0.18 | -0.18 | -0.18 | -0.18 | -0.18 | -0.19 |
|  | (*Hylocichla mustelina*) |  |  |  |  |  |  |  |  |  |  |
| Forest-dependent | Swamp Sparrow | -0.36 | -0.29 | -0.22 | -0.19 | -0.16 | -0.15 | -0.15 | -0.15 | -0.17 | -0.17 |
|  | (*Melospiza georgiana*) |  |  |  |  |  |  |  |  |  |  |
| Forest-dependent | Northern Flicker | -0.32 | -0.24 | -0.21 | -0.20 | -0.17 | -0.16 | -0.16 | -0.15 | -0.15 | -0.14 |
|  | (*Colaptes auratus*) |  |  |  |  |  |  |  |  |  |  |
| Forest-dependent | Great Crested Flycatcher | -0.34 | -0.27 | -0.24 | -0.21 | -0.18 | -0.16 | -0.16 | -0.15 | -0.14 | -0.14 |
|  | (*Myiarchus crinitus*) |  |  |  |  |  |  |  |  |  |  |
| Forest-dependent | Tree Swallow | -0.37 | -0.30 | -0.26 | -0.22 | -0.19 | -0.18 | -0.17 | -0.16 | -0.15 | -0.14 |
|  | (*Tachycineta bicolor*) |  |  |  |  |  |  |  |  |  |  |
| Forest-independent | Field Sparrow | -0.34 | -0.28 | -0.23 | -0.21 | -0.20 | -0.19 | -0.17 | -0.16 | -0.15 | -0.15 |
|  | (*Spizella pusilla*) |  |  |  |  |  |  |  |  |  |  |
| Forest-independent | Northern Rough-winged Swallow | -0.41 | -0.32 | -0.29 | -0.22 | -0.20 | -0.16 | -0.18 | -0.17 | -0.18 | -0.16 |
|  | (*Stelgidopteryx serripennis*) |  |  |  |  |  |  |  |  |  |  |
| Forest-independent | Yellow-breasted Chat | -0.30 | -0.24 | -0.22 | -0.22 | -0.18 | -0.18 | -0.21 | -0.20 | -0.19 | -0.15 |
|  | (*Icteria virens*) |  |  |  |  |  |  |  |  |  |  |
| Forest-independent | Blue Jay | -0.33 | -0.27 | -0.24 | -0.22 | -0.21 | -0.20 | -0.19 | -0.19 | -0.18 | -0.18 |
|  | (*Cyanocitta cristata*) |  |  |  |  |  |  |  |  |  |  |
| Forest-independent | Brown Thrasher | -0.37 | -0.30 | -0.27 | -0.26 | -0.23 | -0.23 | -0.21 | -0.20 | -0.19 | -0.17 |
|  | (*Toxostoma rufum*) |  |  |  |  |  |  |  |  |  |  |
| Forest-independent | Henslow's Sparrow | -0.40 | -0.35 | -0.31 | -0.23 | -0.19 | -0.20 | -0.20 | -0.22 | -0.19 | -0.25 |
|  | (*Ammodramus henslowii*) |  |  |  |  |  |  |  |  |  |  |
| Forest-independent | Grasshopper Sparrow | -0.44 | -0.35 | -0.31 | -0.26 | -0.23 | -0.22 | -0.21 | -0.19 | -0.19 | -0.19 |
|  | (*Ammodramus savannarum*) |  |  |  |  |  |  |  |  |  |  |
| Forest-independent | Alder Flycatcher | -0.34 | -0.27 | -0.24 | -0.24 | -0.24 | -0.23 | -0.23 | -0.20 | -0.20 | -0.19 |
|  | (*Empidonax alnorum*) |  |  |  |  |  |  |  |  |  |  |
| Forest-independent | Blue-winged Warbler | -0.29 | -0.21 | -0.20 | -0.21 | -0.20 | -0.22 | -0.22 | -0.23 | -0.23 | -0.24 |
|  | (*Vermivora cyanoptera*) |  |  |  |  |  |  |  |  |  |  |
| Forest-independent | Downy Woodpecker | -0.34 | -0.28 | -0.26 | -0.24 | -0.22 | -0.21 | -0.21 | -0.21 | -0.21 | -0.21 |
|  | (*Picoides pubescens*) |  |  |  |  |  |  |  |  |  |  |
| Forest-independent | Baltimore Oriole | -0.37 | -0.31 | -0.28 | -0.25 | -0.24 | -0.24 | -0.24 | -0.24 | -0.23 | -0.22 |
|  | (*Icterus galbula*) |  |  |  |  |  |  |  |  |  |  |
| Forest-independent | American Goldfinch | -0.38 | -0.31 | -0.28 | -0.27 | -0.25 | -0.24 | -0.23 | -0.23 | -0.22 | -0.21 |
|  | (*Spinus tristis*) |  |  |  |  |  |  |  |  |  |  |
| Forest-independent | Eastern Bluebird | -0.41 | -0.34 | -0.30 | -0.28 | -0.25 | -0.25 | -0.24 | -0.23 | -0.22 | -0.20 |
|  | (*Sialia sialis*) |  |  |  |  |  |  |  |  |  |  |
| Forest-independent | Gray Catbird | -0.38 | -0.32 | -0.29 | -0.28 | -0.26 | -0.26 | -0.26 | -0.25 | -0.25 | -0.25 |
|  | (*Dumetella carolinensis*) |  |  |  |  |  |  |  |  |  |  |
| Forest-independent | Song Sparrow | -0.40 | -0.33 | -0.30 | -0.29 | -0.27 | -0.26 | -0.25 | -0.25 | -0.24 | -0.22 |
|  | (*Melospiza melodia*) |  |  |  |  |  |  |  |  |  |  |
| Forest-independent | Yellow Warbler | -0.39 | -0.33 | -0.29 | -0.29 | -0.28 | -0.27 | -0.27 | -0.26 | -0.25 | -0.24 |
|  | (*Setophaga petechia*) |  |  |  |  |  |  |  |  |  |  |
| Forest-independent | Orchard Oriole | -0.33 | -0.30 | -0.30 | -0.28 | -0.27 | -0.28 | -0.29 | -0.28 | -0.27 | -0.26 |
|  | (*Icterus spurius*) |  |  |  |  |  |  |  |  |  |  |
| Forest-independent | Bobolink | -0.44 | -0.36 | -0.31 | -0.30 | -0.28 | -0.26 | -0.26 | -0.24 | -0.22 | -0.21 |
|  | (*Dolichonyx oryzivorus*) |  |  |  |  |  |  |  |  |  |  |
| Forest-independent | Mourning Dove | -0.40 | -0.34 | -0.31 | -0.29 | -0.27 | -0.27 | -0.26 | -0.25 | -0.25 | -0.24 |
|  | (*Zenaida macroura*) |  |  |  |  |  |  |  |  |  |  |
| Forest-independent | Barn Swallow | -0.43 | -0.36 | -0.32 | -0.30 | -0.28 | -0.27 | -0.25 | -0.25 | -0.23 | -0.21 |
|  | (*Hirundo rustica*) |  |  |  |  |  |  |  |  |  |  |
| Forest-independent | American Robin | -0.40 | -0.34 | -0.31 | -0.30 | -0.28 | -0.27 | -0.27 | -0.26 | -0.25 | -0.24 |
|  | (*Turdus migratorius*) |  |  |  |  |  |  |  |  |  |  |
| Forest-independent | Eastern Meadowlark | -0.45 | -0.37 | -0.33 | -0.31 | -0.29 | -0.28 | -0.27 | -0.25 | -0.24 | -0.22 |
|  | (*Sturnella magna*) |  |  |  |  |  |  |  |  |  |  |
| Forest-independent | Brown-headed Cowbird | -0.40 | -0.34 | -0.32 | -0.30 | -0.29 | -0.29 | -0.29 | -0.28 | -0.28 | -0.28 |
|  | (*Molothrus ater*) |  |  |  |  |  |  |  |  |  |  |
| Forest-independent | White-eyed Vireo | -0.37 | -0.29 | -0.28 | -0.28 | -0.30 | -0.31 | -0.30 | -0.30 | -0.32 | -0.36 |
|  | (*Vireo griseus*) |  |  |  |  |  |  |  |  |  |  |
| Forest-independent | Red-winged Blackbird | -0.44 | -0.37 | -0.34 | -0.32 | -0.31 | -0.30 | -0.29 | -0.28 | -0.27 | -0.25 |
|  | (*Agelaius phoeniceus*) |  |  |  |  |  |  |  |  |  |  |
| Forest-independent | Northern Cardinal | -0.41 | -0.35 | -0.33 | -0.32 | -0.32 | -0.32 | -0.31 | -0.31 | -0.31 | -0.31 |
|  | (*Cardinalis cardinalis*) |  |  |  |  |  |  |  |  |  |  |
| Forest-independent | House Wren | -0.43 | -0.38 | -0.35 | -0.34 | -0.33 | -0.32 | -0.32 | -0.31 | -0.30 | -0.30 |
|  | (*Troglodytes aedon*) |  |  |  |  |  |  |  |  |  |  |
| Forest-independent | Red-bellied Woodpecker | -0.42 | -0.37 | -0.34 | -0.33 | -0.32 | -0.32 | -0.33 | -0.33 | -0.33 | -0.34 |
|  | (*Melanerpes carolinus*) |  |  |  |  |  |  |  |  |  |  |
| Forest-independent | Killdeer | -0.46 | -0.40 | -0.38 | -0.36 | -0.34 | -0.33 | -0.32 | -0.31 | -0.30 | -0.28 |
|  | (*Charadrius vociferus*) |  |  |  |  |  |  |  |  |  |  |
| Forest-independent | Common Grackle | -0.45 | -0.40 | -0.37 | -0.36 | -0.34 | -0.33 | -0.32 | -0.31 | -0.30 | -0.29 |
|  | (*Quiscalus quiscula*) |  |  |  |  |  |  |  |  |  |  |
| Forest-independent | Chimney Swift | -0.43 | -0.38 | -0.36 | -0.36 | -0.36 | -0.35 | -0.34 | -0.34 | -0.33 | -0.32 |
|  | (*Chaetura pelagica*) |  |  |  |  |  |  |  |  |  |  |
| Forest-independent | Eastern Kingbird | -0.47 | -0.40 | -0.37 | -0.36 | -0.34 | -0.33 | -0.33 | -0.33 | -0.33 | -0.32 |
|  | (*Tyrannus tyrannus*) |  |  |  |  |  |  |  |  |  |  |
| Forest-independent | European Starling | -0.47 | -0.42 | -0.39 | -0.37 | -0.34 | -0.34 | -0.33 | -0.32 | -0.31 | -0.30 |
|  | (*Sturnus vulgaris*) |  |  |  |  |  |  |  |  |  |  |
| Forest-independent | Savannah Sparrow | -0.50 | -0.43 | -0.41 | -0.37 | -0.36 | -0.35 | -0.34 | -0.33 | -0.31 | -0.27 |
|  | (*Passerculus sandwichensis*) |  |  |  |  |  |  |  |  |  |  |
| Forest-independent | Carolina Wren | -0.42 | -0.38 | -0.37 | -0.37 | -0.37 | -0.37 | -0.37 | -0.37 | -0.37 | -0.38 |
|  | (*Thryothorus ludovicianus*) |  |  |  |  |  |  |  |  |  |  |
| Forest-independent | Warbling Vireo | -0.47 | -0.41 | -0.38 | -0.38 | -0.38 | -0.37 | -0.36 | -0.36 | -0.35 | -0.35 |
|  | (*Vireo gilvus*) |  |  |  |  |  |  |  |  |  |  |
| Forest-independent | Rock Pigeon | -0.55 | -0.47 | -0.45 | -0.42 | -0.38 | -0.37 | -0.35 | -0.33 | -0.30 | -0.29 |
|  | (*Columba livia*) |  |  |  |  |  |  |  |  |  |  |
| Forest-independent | Willow Flycatcher | -0.46 | -0.40 | -0.38 | -0.39 | -0.40 | -0.39 | -0.38 | -0.37 | -0.36 | -0.35 |
|  | (*Empidonax traillii*) |  |  |  |  |  |  |  |  |  |  |
| Forest-independent | House Sparrow | -0.50 | -0.45 | -0.44 | -0.42 | -0.41 | -0.40 | -0.39 | -0.38 | -0.37 | -0.36 |
|  | (*Passer domesticus*) |  |  |  |  |  |  |  |  |  |  |
| Forest-independent | House Finch | -0.50 | -0.46 | -0.44 | -0.43 | -0.42 | -0.41 | -0.40 | -0.40 | -0.40 | -0.39 |
|  | (*Haemorhous mexicanus*) |  |  |  |  |  |  |  |  |  |  |
| Forest-independent | Northern Mockingbird | -0.51 | -0.49 | -0.48 | -0.48 | -0.47 | -0.47 | -0.46 | -0.47 | -0.47 | -0.46 |
|  | (*Mimus polyglottos*) |  |  |  |  |  |  |  |  |  |  |
| Forest-independent | Carolina Chickadee | -0.52 | -0.50 | -0.52 | -0.54 | -0.57 | -0.60 | -0.61 | -0.62 | -0.63 | -0.64 |
|  | (*Poecile carolinensis*) |  |  |  |  |  |  |  |  |  |  |
