# Supplementary material for: The traits that predict the magnitude and spatial scale of forest bird responses to urbanization intensity
Source: PLoS One. 2019 Jul 25;14(7):e0220120. doi: 10.1371/journal.pone.0220120 (PMC6657869; doi:10.1371/journal.pone.0220120)
Supplement: S2 Table — (DOCX) [file pone.0220120.s006.docx]

**S2 Table.** Results from general linear models of the effects of traits on forest bird species’ responses to urbanization intensity in Pennsylvania, USA.

| Trait | λ ML | 95% CI | *β* | Residual df | Adjusted R^2^ |
| --- | --- | --- | --- | --- | --- |
| Biparental nestbuilding | 0 | NA - 0.28 | 16.56 | 54 | -0.01 |
| Body mass | 0 | NA - 0.28 | 0.05 | 56 | -0.01 |
| Cavity nesting | 0 | NA - 0.25 | 51.47 | 56 | 0.02 |
| Clutch size | 0 | NA - 0.29 | 21.80 | 56 | 0.03 |
| Clutches per year | 0 | NA - 0.33 | 25.79 | 50 | -0.01 |
| Duration in nest | 0 | NA - 0.29 | 1.69 | 54 | -0.01 |
| Fledglings per nest | 0 | NA - 0.42 | 26.78 | 28 | 0.08 |
| Flock size | 0 | NA - 0.38 | 0.56 | 32 | -0.03 |
| Foraging height | 0 | NA - 0.26 | -2.19 | 38 | -0.01 |
| Frugivory | 0 | NA - 0.36 | 34.18 | 56 | 0.00 |
| Granivory | 0 | NA - 0.21 | 64.84 | 56 | 0.06 |
| Lifespan | 0 | NA - 0.27 | 3.98 | 54 | -0.01 |
| Migratory status | 0 | NA - 0.20 | 56.41 | 56 | 0.03 |
| Nesting height | 0 | NA - 0.30 | 1.68 | 51 | -0.01 |
| Omnivory | 0 | NA - 0.28 | 43.10 | 56 | 0.01 |
| Song frequency | 0 | NA - 0.30 | -0.01 | 47 | -0.02 |
| Song length | 0 | NA - 0.28 | 1.95 | 47 | -0.02 |
| Song range | 0 | NA - 0.36 | -0.03 | 47 | -0.02 |
| Territory | 0 | NA - 0.79 | 0.00 | 36 | -0.03 |
| Wingspan | 0 | NA - 0.39 | 0.58 | 54 | -0.01 |
